# The influence of passivation and photovoltaic properties of α-Si:H coverage on silicon nanowire array solar cells

**DOI:** 10.1186/1556-276X-8-396

**Published:** 2013-09-23

**Authors:** KunTang Li, XiuQin Wang, PengFei Lu, JianNing Ding, NingYi Yuan

**Affiliations:** 1 Center for Low-Dimensional Materials, Micro-Nano Devices and Systems, Jiangsu Key Laboratory for Solar Cell Materials and Technology, Changzhou University, Changzhou, Jiangsu 213164, China; 2 Jiangsu Collaborative Innovation Center of Photovolatic Science and Engineering, Changzhou, Jiangsu 213164, China; 3 Center of Micro/Nano Science & Technology, Jiangsu University, Zhenjiang 212013, China

**Keywords:** Radial p-n SiNW solar cell, Hydrogenated amorphous silicon, Surface coverage, Open circuit voltage

## Abstract

Silicon nanowire (SiNW) arrays for radial p-n junction solar cells offer potential advantages of light trapping effects and quick charge collection. Nevertheless, lower open circuit voltages (*V*_oc_) lead to lower energy conversion efficiencies. In such cases, the performance of the solar cells depends critically on the quality of the SiNW interfaces. In this study, SiNW core-shell solar cells have been fabricated by growing crystalline silicon (c-Si) nanowires via the metal-assisted chemical etching method and by depositing hydrogenated amorphous silicon (α-Si:H) via the plasma-enhanced chemical vapor deposition (PECVD) method. The influence of deposition parameters on the coverage and, consequently, the passivation and photovoltaic properties of α-Si:H layers on SiNW solar cells have been analyzed.

## Background

Nanowire-based solar cells hold promise for next generation photovoltaics. In particular, silicon micro/nanowires have attracted considerable interest due to their potential advantages, including light trapping effects to enhance broadband optical absorption [1,2] and the possibility to engineer radial p-n junctions using a core-shell structure, which in turn increases the carrier collection [3-14]. In a radial p-n junction - a promising approach - crystalline silicon (c-Si) micro/nanowires are used as core and high-temperature diffused layers or low-temperature deposited silicon layers form the shell. These core-shell micro/nanowire array structures are expected to reduce the requirements on the quality and the quantity of Si needed for the fabrication of solar cell.

Thus far, several methods have been established for the controlled growth of silicon nanowires (SiNWs). For instance, highly parallel SiNWs of desired lengths and diameters ranging from a few tens of nanometers to a few hundreds of nanometers could conventionally be obtained by aqueous electroless chemical etching of single crystalline silicon wafers [15-20]. Similarly, hydrogenated amorphous silicon (α-Si:H) can be deposited by the plasma-enhanced chemical vapor deposition (PECVD) method. According to this report, an efficiency of 7.29% was realized by fabricating a core-shell nanowire solar cell with the structure TCO/α-Si:H (p^+^)/α-Si:H (i)/c-Si (n) [13]. In addition, it has been demonstrated that the deposition of an ultrathin passivating Al_2_O_3_ tunnel layer on the highly doped p-type α-Si:H, prior to the deposition of TCO, further increases the efficiency to 10.0% [14].

However, there are certain shortcomings that need to be addressed to fabricate nanowire solar cells with expected efficiency. For example, a low open circuit voltage (*V*_oc_) in SiNW solar cells results in low energy conversion efficiency compared to the efficiency of bulk Si solar cells. Moreover, compared to Si microwire (SiMW) solar cells [5-8], which are formed by deep reactive ion etching, the *V*_oc_ of SiNW solar cells is typically lower. This could be attributed to the large surface-to-volume ratio exhibited by SiNWs. Essentially, the performance of SiNW solar cells depends critically on the quality of the SiNW interfaces. Hence, surface passivation of SiNWs is a critical process for solar cell applications. Compared with the fabrication of planar c-Si and Si microwire arrays, surface passivation of SiNWs is a more challenging task due to the small size and the possible bundling of NWs [15-20]. Some reports have demonstrated high-efficiency silicon photovoltaics through excellent surface passivation of crystalline planar Si using α-Si:H deposited by PECVD [21-23]. Nevertheless, to the best of our knowledge, there are not many systematic studies on the deposition of α-Si:H, and reports analyzing the influence of thickness and coverage of this amorphous silicon layer on the surface passivation as well as the open circuit voltage of the fabricated cells.

Hence, in this work, we have prepared SiNWs using metal-assisted chemical etching method and deposited α-Si:H passivation layers by PECVD method. Furthermore, we have studied the effect of PECVD deposition conditions of α-Si:H, such as plasma power and deposition time, on the coverage of α-Si:H layers on SiNWs. In addition, we have evaluated the influence of passivation quality and thickness of α-Si:H layers on the open circuit voltage of the fabricated silicon nanowire array solar cells.

## Methods

### Treatment of the backside of Si wafers

In this study, double side polished p-type solar grade Si (100) wafers of thickness 180 μm and resistivity 1 to 2 Ω cm were used for the fabrication of solar cells. Prior to fabrication, Si wafers were initially cleaned in a solution of NH_4_OH/H_2_O_2_/H_2_O (1:1:5), followed by cleaning in a boiling solution of HCl/H_2_O_2_/H_2_O (1:1:5). The cleaned wafers were subsequently immersed in dilute HF solution to remove surface oxides and finally dried in a flux of nitrogen. Starting with the cleaned Si wafers, the layers to be deposited on the backside of the Si wafers were fabricated before the growth of SiNWs.

In order to measure the effective lifetime of as-prepared SiNWs and α-Si:H-covered SiNWs, 25-nm-thick Al_2_O_3_ layers were deposited on the backside of the wafers by the atomic layer deposition (ALD) method. The Si wafers thus obtained were subsequently annealed at 400°C in N_2_/H_2_ for 10 min to passivate the backside of the Si wafers. For this, trimethylaluminum (TMA, Al(CH_3_)_3_) and water (H_2_O) were used as precursors. High-purity nitrogen (N_2_) gas was used as the carrier and purge gas. Processing temperature and pressure were set to 200°C and 100 Pa, respectively.

Further, another backside treatment was adopted to fabricate the SiNW solar cells. Al paste (Dupont 1287, Wilmington, DE, USA) was coated on the backside of the Si wafers, which were finally annealed at 850°C for 1 min in N_2_ atmosphere.

### Preparation of silicon nanowire array

Following the treatments on the backside of the Si wafers, vertically aligned SiNWs were grown on the other side (front side) of the Si wafers by the metal-assisted chemical etching method. This involved the electroless deposition of Ag particles in AgNO_3_/HF solution and subsequent Ag-assisted etching in the same solution. During the chemical etching process, the backside of the Si wafers with Al_2_O_3_ or Al layers was protected using a Teflon container. In the typical process, the etchant containing silver ions (Ag^+^, 0.02 M) and fluoric acid (HF, 5.0 M) was used for the growth of SiNWs. Etching time was controlled at 3 and 5 min to obtain SiNWs of desired dimension at 50°C. After etching, the as-prepared samples were immersed in 50% conc. HNO_3_ and 5% conc. HF, successively, to remove residual Ag particles and SiO_2_. Finally, the samples were rinsed with deionized water and dried at room temperature in a smooth nitrogen flux.

### Deposition of α-Si:H layers and fabrication of silicon nanowire array solar cells

Subsequently, α-Si:H layers were deposited by radio frequency PECVD method. Prior to the deposition of α-Si:H, the SiNWs prepared by chemical etching were exposed to H_2_ plasma at a plasma power of 30 W for 1 min to clean the surface in a PECVD chamber. For the intrinsic growth of α-Si:H layers, 10 sccm of 5% H_2_-diluted SiH_4_ was introduced in the PECVD chamber, while maintaining a substrate temperature of 180°C and a pressure of 100 Pa. To fabricate SiNW solar cells, a mixture of 10 sccm of 5% H_2_-diluted SiH_4_, 1 sccm of 0.5% H_2_-diluted PH_3_, and 40 sccm of H_2_ was introduced for 20 min to deposit n-type Si:H layers above intrinsic α-Si:H layers. During the deposition, the substrate temperature was maintained at 180°C, at a pressure of 150 Pa and power of 70 W. Following that, 3% Al-doped ZnO (AZO) films were deposited on the as-grown n-type Si:H layers by ALD method. For that, diethyl zinc (DEZ), TMA, and water were used as precursors, and the deposition was performed at 200°C for 1 h, resulting in the formation of 90-nm-thick Al-doped ZnO films. Finally, Ag grid electrodes of thickness 100 nm were deposited by sputtering method using a mask.

### Characterization

The surface and cross-sectional morphology of the prepared SiNWs were analyzed using field emission scanning electron microscope (FESEM) (Philips XL30 FEG, FEI, Hillsboro, OR, USA) and transmission electron microscope (TEM, JEOL JEM-2100, Akishima, Tokyo, Japan). For TEM analysis, SiNWs were scratched from the silicon substrates and dispersed in ethanol by ultrasonic. The antireflection properties of SiNW arrays were evaluated by reflectivity measurement under UV-visible light absorption. The effective lifetimes (*τ*_eff_) were investigated using microwave-detected photoconductance decay (μPCD) technique [24]. The extraction of *τ*_eff_ within a semiconductor sample by means of the μPCD measurement method is based on the change of the reflectance of a microwave when irradiated on the sample. A short laser pulse, with a constant pulse width of *t*_p_ = 200 ns optically generated excess charge carriers. This change of the excess charge carrier density is directly linked with a change of the conductivity of the sample. After the laser is switched off, the conductivity decreases monoexponentially and can be fitted with an exponential curve to extract the effective lifetime at a given position of the sample. The measurement setup used in this contribution is the commercially available WT-2000 tool distributed by Semilab Semiconductor Physics Laboratory Co. Ltd., Budapest, Hungary.

### Photovoltaic measurements

Photovoltaic parameters of the fabricated SiNW array solar cell, namely open circuit voltage (*V*_oc_) and short circuit current density (*J*_sc_), were measured using a Keithley 2400 source meter (Cleveland, OH, USA). A solar simulator (500-W Xe lamp) was employed as the light source, and incident light intensity was calibrated using a standard silicon solar cell and light intensity meter (Radiometer FZ-A, Copenhagen, Denmark), simultaneously. The external quantum efficiency (EQE) experiments were carried out using a system consisting of a Xe lamp (300 W) with a monochromator (Oriel 74100, Newport Corp., Irvine, CA, USA). The light intensity was measured with an optical power meter (Ophir Optronics 70310, Newport Corp.) equipped with a calibrated thermopile head (Ophir Optronics 71964, Newport Corp.).

## Results and discussion

### Characterization of as-deposited and α-Si:H-covered silicon nanowire arrays

**Figure 1:**
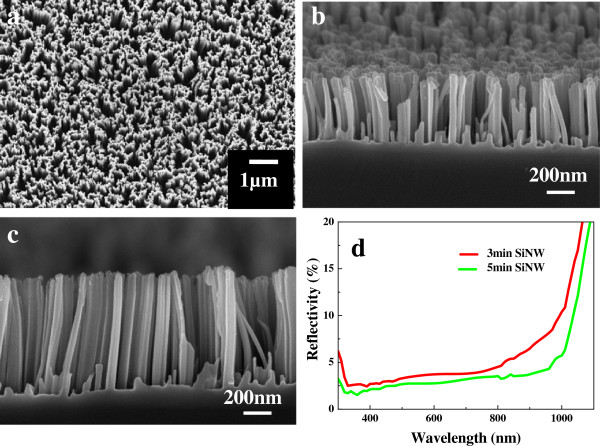
**FESEM images.** The top view **(a)** and cross-sectional views **(b, c)** and reflectance spectra **(d)** of the SiNWs etched for 3 and 5 min.

The typical top view FESEM image of the as-deposited SiNW array (Figure 1a) indicates the formation of a uniform surface. However, some SiNWs are observed to form congregated bundles. The cross-sectional FESEM images of the SiNWs grown by etching for 3 and 5 min at 50°C, as shown in Figure 1b,c, respectively, indicate straight growth of nanowires vertical to the substrate, resulting in a smooth surface with almost no pores. The typical length of the SiNWs obtained by etching for 3 and 5 min is estimated to be approximately 0.51 and approximately 0.85 μm, respectively. The diameters range from tens of nanometers up to 200 nm, while the distance between the adjacent NWs range from several tens of nanometers up to approximately 300 nm. The reflectance spectra of the SiNWs grown by etching for 3 min (Figure 1d) indicate a reflectivity of about 3% in the wavelength range of 300 to 800 nm. At wavelengths larger than 800 nm, the reflectivity shows a slight increase. When the etching time is extended to 5 min, the reflectivity is further decreased, especially in the wavelength range of 800 to 1,000 nm.

**Figure 2:**
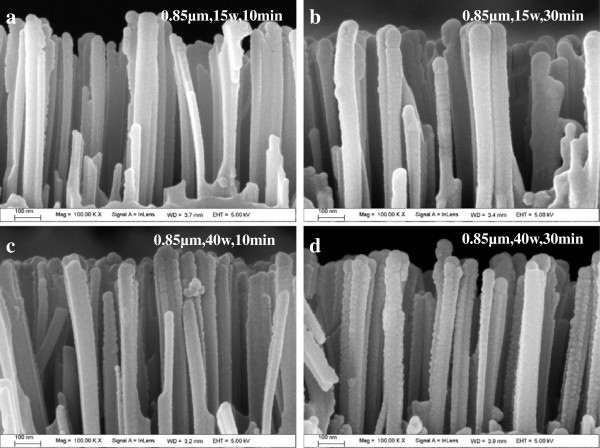
**Cross-sectional FESEM views (a to d) of the 0.85-μm SiNWs after deposition of α-Si:H passivation layer.** Using plasma power of 15 and 40 W for 10 and 30 min, respectively.

**Figure 3:**
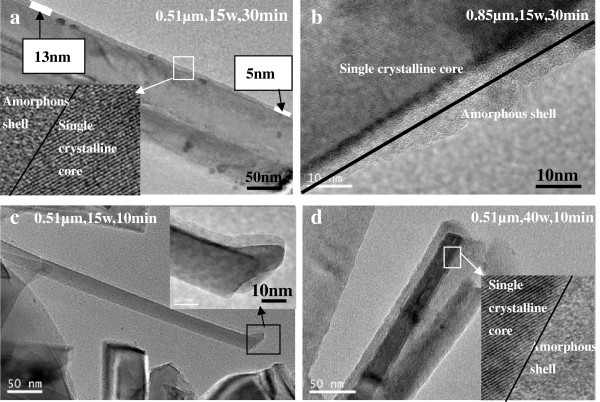
**TEM images (a to d) of SiNWs after deposition of α-Si:H passivation layer.** With a plasma power of 15 and 40 W. The inset high-resolution transmission electron microscope (HRTEM) image of a core-shell silicon nanowire shows that the core is single crystalline while the shell is amorphous.

Figure 2a,b,c,d show the cross-sectional FESEM images of the 0.85-μm SiNWs (5-min-etched SiNWs) shown in Figure 1c, after the deposition of intrinsic α-Si:H using plasma power of 15 and 40 W for 10 and 30 min, respectively. It can be observed that the thickness of the α-Si:H layer deposited using a plasma power of 40 W is thicker than that deposited at 15 W, which implies that the deposition rate of α-Si:H is much larger at 40 W. Moreover, it can be noticed that the coverage of Si:H layers on the NW walls is not homogeneous along the vertical direction. This is further confirmed using the TEM images shown in Figure 3. As seen from the TEM image of the 0.51-μm SiNW (3-min-etched SiNW) shown in Figure 3a, when the deposition time is 30 min and the plasma power is 15 W, the thickness of α-Si:H layers varies from approximately 13 to approximately 5 nm along the axial direction of the SiNW. However, in the case of 0.85-μm SiNW, the resulting α-Si:H layers barely cover the bottom of the NW completely, as indicated in Figure 3b. When the deposition time is decreased to 10 min, the thickness of α-Si:H layer deposited at 15 W on the top of the SiNW is about approximately 5.6 nm (Figure 3c), while it is approximately 11.8 nm when the deposition is performed at 40 W (Figure 3d). This indicates that the deposition rate of α-Si:H layers at 40 W is twice of that at 15 W. Moreover, the high-resolution TEM images (shown as insets in Figure 3a,d) reveal that the nanowire is composed of a single-crystalline core and amorphous silicon (a-Si) shell. There is no evidence for the formation of crystalline phase or structural defects either at the c-Si/α-Si:H interface or in the α-Si:H bulk. The results clearly substantiate the formation of purely amorphous intrinsic silicon bulk and abrupt c-Si/α-Si:H interface.

**Figure 4:**
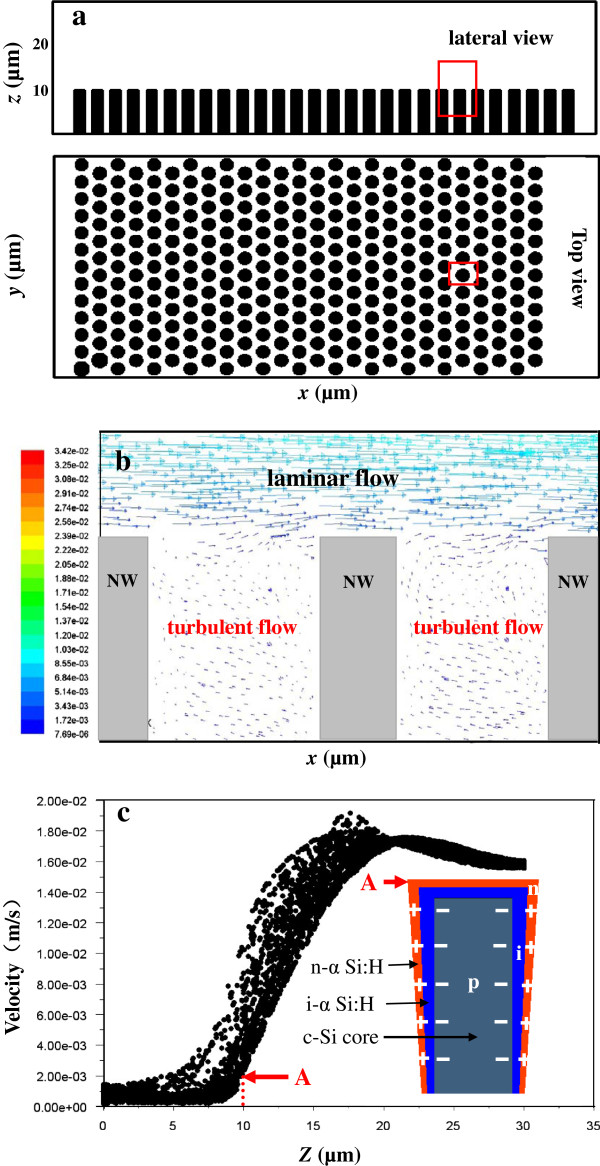
**Schematic of the simulated chamber, simulated velocity vector graphs, and simulated gas velocity. (a)** Schematic of the simulated chamber containing a 14 × 14 SiNW array of diameter 0.2 μm and height 1.0 μm, and at a distance of 0.2 μm between adjacent NWs. **(b)** Simulated velocity vector graphs in the given areas as the red square indicated in **(a)**. A laminar flow above the NW array and a turbulent flow in the gap between the NWs are obtained. **(c)** Simulated gas velocity at the mesh points at the *y-z*-plane along the z-axis. Point A presents the top of NWs. The inset in **(c)** gives the schematic illustrating the coverage of α-Si:H layers on SiNWs and the built-in electrical field.

The cause for the observed non-uniformity in the coverage of α-Si:H layers on SiNWs has been analyzed by computational fluid dynamics (CFD) simulation of gas flow in the NW array. The commercial CFD package, FLUENT, which employs the finite volume method, was used for numerical computations. The schematic sketch of the chamber containing NW array of diameter 0.2 μm and height 1 μm, with a distance of 0.2 μm between the adjacent NWs, is shown in Figure 4a. The flow boundary conditions set the inlet gas velocity to 1 cm s^−1^ at the left vertical wall of the chamber, and the gas was pulled out through the right vertical wall. The pressure in the chamber was set as 100 Pa. A grid containing about 956,465 meshes was used for the numerical computation in this study. The simulated velocity vector graphics (of the region in the red box shown in Figure 4a) in the *x*-*z*-plane is shown in Figure 4b. Although the gas flow in the NW array is completely turbulent, it could be observed that there still exists a laminar flow layer adjacent to the top of the NW array, where the flow velocity is much higher than that in the NW array. Moreover, the velocity drops along the NW sidewall, which is further demonstrated by the simulated velocity of the mesh spots at the *y*-*z*-plane (*x* = 100 mm) along the *z*-axis (NW growth direction) in Figure 4c. This explains the observed experimental results.

During the PECVD process, since the SiNWs are closely packed, the flow velocity of reaction gas is not only much slower in the gaps between the SiNWs than on the planar surface but also is gradually decreased along the vertical direction of SiNWs. Under this condition, the gas in the feed suspension is prone to be deposited on the top surface of the NWs to form a thick layer. This results in inhomogeneous coverage of α-Si:H layers on NW walls along the vertical direction, as shown in the inset in Figure 4c. Hence, a low deposition rate produced by a small plasma power is more favorable to supplement fresh reaction gas at the bottom of SiNWs, consequently to obtain a relatively uniform coverage of a-Si layers.

### Passivation properties of α-Si:H on silicon nanowire arrays

**Figure 5:**
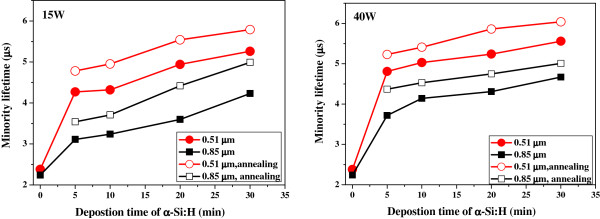
Dependence of minority lifetime of 0.51- and 0.85-μm SiNWs on plasma power and deposition time of α-Si:H.

The measured minority carrier lifetimes (*τ*_eff_) of the as-prepared SiNW arrays and the arrays passivated by α-Si:H layers deposited under different plasma powers for different times are presented in Figure 4. The experimental results indicate a *τ*_eff_ value of 2.24 and 2.38 μs for 3- and 5-min-etched SiNWs, respectively. The effective carrier lifetime is determined by the following equation: 1/*τ*_eff_ = 1/*τ*_b_ + 4*S* / *d*[25], where *S* is the surface recombination rate, *d* denotes the nanowire diameter, and *τ*_b_ represents the carrier lifetime in bulk Si. A large number of surface defects were generated during the growth of the NWs by the metal-assisted chemical etching process. As the surface recombination rate increases in front, the effective lifetime, which is a contribution of bulk and surface lifetimes, decreases for silicon NWs. To suppress the defects generated during the growth of nanowires by chemical etching process, the surface passivation was carried out. As evidenced from Figure 5, the overall *τ*_eff_ values improved after the deposition of α-Si:H passivation layers. In fact, the *τ*_eff_ value increased with the deposition time and deposition power of α-Si:H. The longer deposition time and increased deposition power will in turn increase the relative thickness of α-Si:H passivation layers. The largest *τ*_eff_ value was obtained for 0.51-μm SiNWs passivated at a plasma power of 40 W for 30 min. This indicates that relatively thicker α-Si:H layers are highly favorable to reduce the density of dangling bonds on the SiNW surfaces.

In general, it is believed that the surface passivation properties of the α-Si:H layer greatly improves upon additional thermal annealing at certain temperatures. However, the annealing temperature should not be too high in order to prevent escape of H in α-Si:H. On the basis of this reason, the annealing temperature was chosen as 200°C, and the subsequent preparation of AZO was performed at 200°C. The improvement was quantitatively evaluated by annealing the as-deposited samples at 200°C for 1 h in N_2_ ambient. As expected, the annealed samples show improvement in the surface passivation properties (Figure 5). This is owing to the fact that additional thermal annealing can facilitate improved hydrogen redistribution to the interface region. Moreover, it has also been reported that atomic hydrogen under thermal treatment can interchange from the easilybroken Si-H_2_ bonds existing near the c-Si/a-Si:H interface to passivate the dangling bonds. After such thermal treatment, the transformation of Si-H_2_ to Si-H results in effective restructuring for improved surface passivation properties [26].

### Photovoltaic properties of SiNW solar cells

**Figure 6:**
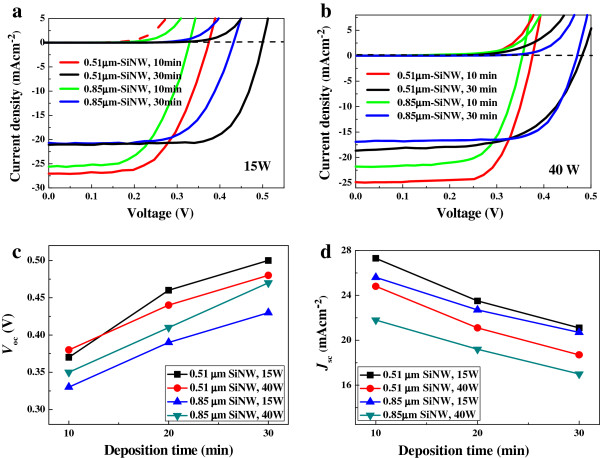
***J*-*V *curves measured in the dark and at AM1.5 illumination for 0.51- and 0.85-μm SiNW solar cells.** With α-Si:H passivation layer deposited at plasma power of 15 W **(a)** and 40 W **(b)**. Dependence of open voltage and short current density plotted as a function of plasma power **(c)** and deposition time **(d)**.

SiNW solar cells were fabricated by depositing n-type α-Si:H layers above the intrinsic α-Si:H layers. Subsequently, 90-nm-thick polycrystalline AZO layers were coated by ALD method, at 200°C for approximately 1 h. The current voltage (*J-V*) measurements of the SiNW solar cells with α-Si:H deposited at 15 and 40 W, respectively, were performed in the dark and at AM1.5 illumination, as shown in Figure 6a,b. The solar cell had an area of 1 cm^2^. As evidenced from the figures, the *J*-*V* curves show a perfect rectifying behavior. No shunting at reverse bias could be observed for all the cells. The measured parameters are summarized in Tables 1 and 2. The dependence of *V*_oc_ and *J*_sc_ on plasma power and deposition time of α-Si:H is depicted in Figure 6c,d. It can be clearly seen that *V*_oc_ increases with increase in deposition time. For 0.85-μm SiNW solar cells, the *V*_oc_ with α-Si:H deposited at 40 W is larger than its counterpart with α-Si:H deposited at 15 W. This could be attributed to the following two reasons. Firstly, with increase in deposition power, the thickness of α-Si:H layers and measured minority lifetimes increase, which reflect a relatively good mean passivation quality of SiNWs. The other reason is that, the *V*_oc_ is also well known to be dependent on the built-in potential of the solar cell structure. For very thin α-Si:H layer, where the band bending in the α-Si:H layer is not completely achieved, *V*_oc_ depends strongly on the thickness. The deposition rate of α-Si:H at 15 W is slower than that at 40 W, as shown in Figures 2 and 3. In particular, for the 0.85-μm SiNW, the thickness of α-Si:H layer deposited at 15 W at the bottom of SiNW tends to be ultrathin, as shown in Figure 3b, which in turn will influence the band bending that consequently determines the built-in field.

**Table 1:** Performance of SiNW solar cells with α-Si:H layers deposited under 15-W plasma power

**SiNW**	**0.51-μm SiNW**				**0.85-μm SiNW**			
Plasma power (W)	15				15			
Deposition time of α-Si:H (min)	0	10	20	30	0	10	20	30
*J* (mA cm^−2^)	22.8	27.3	23.5	21.1	21.0	25.6	22.7	20.7
*V*_oc_ (V)	0.33	0.37	0.46	0.50	0.31	0.33	0.39	0.43
FF	0.61	0.64	0.67	0.67	0.61	0.63	0.67	0.69
*η* (%)	4.59	6.46	7.24	7.07	3.97	5.32	5.93	6.14

**Table 2:** Performance of SiNW solar cells with α-Si:H layers deposited under 40-W plasma power

**SiNW**	**0.51-μm SiNW**				**0.85-μm SiNW**			
Plasma power (W)	40				40			
Deposition time of α-Si:H (min)	0	10	20	30	0	10	20	30
*J* (mAcm^−2^)	22.8	24.8	21.1	18.7	21.0	21.8	19.2	17.0
*V*_oc_ (V)	0.33	0.38	0.44	0.48	0.31	0.35	0.41	0.47
FF	0.61	0.65	0.68	0.69	0.61	0.65	0.66	0.70
*η* (%)	4.59	6.13	6.17	6.19	3.97	4.96	5.20	5.59

However, in the case of 0.51-μm SiNW solar cell, the dependence of *V*_oc_ on plasma power seems to be contrary. Due to the shorter length, the thickness of α-Si:H layer deposited at the bottom of 0.51-μm SiNW is much larger than that deposited on 0.85-μm SiNW. In addition to the passivation effect, variation in α-Si:H layer thickness on SiNWs along the vertical direction is expected to influence the *V*_oc_. The variation modulates the depletion region of the radial p-n junction, which makes the distribution of built-in electric field in SiNW radial p-n junction uneven, as shown in the inset in Figure 3c. Due to the inhomogeneity of α-Si:H coverage, the SiNW cell performs analogous to solar cells in parallel and consequently leads to a low voltage. From the simulation, it can be expected that low plasma power will result in uniform coverage. Although the measured minority lifetimes are shorter for the SiNW array with α-Si:H deposited at 15 W than those at 40 W, the largest *V*_oc_ of 0.50 V was observed for 0.51-μm SiNW passivated at 15 W for 30 min. The largest *V*_oc_ of 0.50 V is similar to the results obtained from the nanowire device demonstrated by Jia et al. [13,14]. Nevertheless, the observed *V*_oc_ value is still lower than that of SiMW solar cells [5-8]. It is suggested that the inhomogeneity of α-Si:H coverage and passivation on SiNWs along the vertical direction reduces the open circuit voltage.

**Figure 7:**
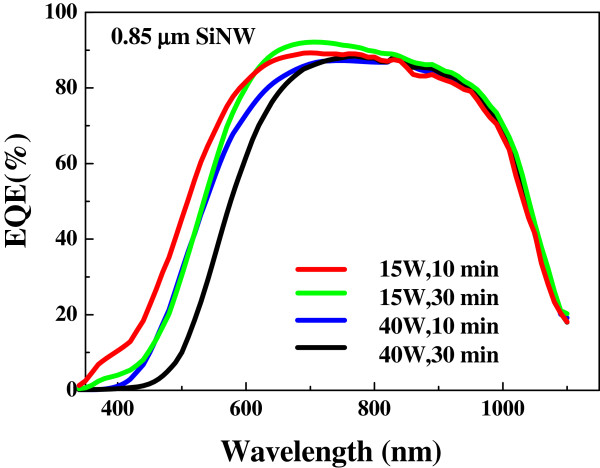
Comparison of external quantum efficiency of 0.85-μm SiNW solar cells.

On the other hand, the dependence of *J*_sc_ on deposition time of α-Si:H is opposite to *V*_oc_, as shown in Figure 5d. It was observed that the prolonged deposition time decreases the current density, which could be ascribed to the increase in the thickness of α-Si:H layers. It is always expected that the nanowire surface passivation is only required for very thin conformal shell layer [14], in which the thicker amorphous shell may contribute to the higher resistance, degrading the carrier collection efficiency, parallel to the passivation of the nanowire surface dangling bonds. Although the reflectance measurement indicates that the 0.85-μm SiNW array has a lower reflectance, which means to have a more light trapping effect, the largest *J*_sc_ was achieved for the 0.51-μm SiNW. Therefore, high photovoltaic conversion efficiency (PCE) was achieved in 0.51-μm SiNW solar cell with α-Si:H deposited at a power of 15 W for 20 min. Comparison of EQE of the 0.85-μm SiNW cells is shown in Figure 7, which further illustrates the effect of α-Si:H coverage. EQE in the wavelength range of 700 to 1,100 nm is nearly the same for the four cells constructed in this study. However, EQE in the wavelength range of 400 to 600 nm shows a remarkable decrease with the increase of plasma power and deposition time.

## Conclusion

In this work, we have analyzed the influence of deposition conditions and surface passivation properties of α-Si:H layer on the nanowire arrays. The thickness of α-Si:H layer and minority lifetime of the SiNW array was found to increase with the increase of deposition time and plasma power. The open circuit voltages of 0.85-μm SiNW solar cells increase with the deposition time and plasma power, while the open circuit voltage dependence of 0.51-μm SiNW solar cells seems to be contrary. The largest *V*_oc_ of 0.50 V was observed for the 0.51-μm SiNW solar cell with α-Si:H passivation layer deposited at 15 W for 30 min. During the PECVD process, since the SiNWs were closely packed, the coverage of α-Si:H layer is inhomogeneous. It is suggested that the open circuit voltage not only depends on the thickness and coverage of the amorphous silicon layer but also on the inhomogeneity of amorphous silicon coverage. The inhomogeneity of α-Si:H coverage and passivation on SiNWs along the vertical direction would lead to a low open circuit voltage and consequently low efficiency of SiNW solar cells.

## Competing interests

The authors declare that they have no competing interests.

## Authors’ contributions

JD and NY conceived and designed the experiments and wrote the paper. KL carried out the experiments and took part in writing the manuscript. XW and FL participated in the experiments. All authors read and approved the final manuscript.
